# Safety and efficacy of compound methyl salicylate liniment for topical pain: A multicenter real-world study in China

**DOI:** 10.3389/fphar.2022.1015941

**Published:** 2022-10-21

**Authors:** Jie Guo, Xiaolei Hu, Jing Wang, Bin Yu, Juan Li, Jianting Chen, Xiaoli Nie, Zhijian Zheng, Shixuan Wang, Qun Qin

**Affiliations:** ^1^ National Institution of Drug Clinical Trial, Xiangya Hospital, Central South University, Changsha, China; ^2^ National Clinical Research Center for Geriatric Disorders, Xiangya Hospital, Central South University, Changsha, China; ^3^ Phase I Clinical Research Center, Xiangya Hospital, Central South University, Changsha, China; ^4^ Hunan Provincial People’s Hospital, The First Affiliated Hospital of Hunan Normal University, Changsha, China; ^5^ Nanfang Hospital, Southern Medical University, Guangzhou, China; ^6^ Beijing Hospital, Beijing, China; ^7^ The Second Affiliated Hospital of Liaoning University of Traditional Chinese Medicine, Shenyang, China; ^8^ International Science and Technology Innovation Cooperation Base for Early Clinical Trials of Biological Agents in Hunan Province, Changsha, China

**Keywords:** ammeltz, soft tissue injury, visual analog scale, new drug monitoring, adverse drug reaction

## Abstract

Compound methyl salicylate liniment (Ammeltz) is composed of various components, such as methyl salicylate, menthol, camphor, chlorpheniramine maleate, and thymol. It was approved for listing in China in 2011. The purpose of this phase Ⅳ clinical trial was to evaluate the safety and efficacy of Ammeltz in a real-life environment in China. Adverse events and adverse drug reactions were used to assess the safety of the monitored drugs. Visual analog scale (VAS) scores were evaluated to assess the severity of pain and the pain relief rate was used to evaluate the efficacy of the study drug. Of 3,600 subjects enrolled, 3,515 (97.64%) subjects completed the study and 85 (2.36%) terminated the study prematurely. A total of 277 adverse events occurred in 258 subjects (7.28%). The most common adverse events included upper respiratory infections (130 cases, 3.67%), local pruritus (17 cases, 0.48%), and diarrhea (12 cases, 0.34%). A total of 50 (1.41%) subjects experienced 58 adverse drug reactions. The most common adverse drug reactions included local pruritus (17 cases, 0.48%), a burning sensation at the application site (10 cases, 0.28%), and irritation at the application site (local) (7 cases, 0.2%). No adverse reactions were identified as new adverse drug reactions. The majority of adverse drug reactions were mild (48 cases, 1.36%), and no severe adverse drug reactions occurred. The subjects experienced significant pain relief after using Ammeltz (mean VAS scores: 5.34 *vs*. 2.79; Day 7 ± 1 *vs*. Baseline; *p* < 0.0001). The pain relief rate was 47.11% ± 23.13%, and in 2,769 cases (78.31%) the drug was effective in pain relief. After excluding subjects who used drugs that could affect the efficacy of the study drug, the subgroups of subjects experienced significant pain relief after using Ammeltz (mean VAS scores: 5.31 vs 2.77; Day 7 ± 1 vs Baseline; *p* < 0.0001). The pain relief rate was 47.34% ± 23.00%, and 2,612 subjects (78.75%) experienced effective pain relief. In conclusion, Ammeltz is safe and effective in real-life use. It can significantly relieve soft tissue pain caused by shoulder and neck pain, back pain, or muscle pain. No new adverse drug reactions were found in our multicenter real-world study.

**Clinical Trial Registration:** https://clinicaltrials.gov/ct2/show/NCT05489939?cond=Safety+and+efficacy+of+compound+methyl+salicylate+liniment+for+topical+pain%3A+A+multicenter+real-world+study+in+China&draw=2&rank=1, identifier NCT05489939

## Introduction

Pain is considered the fifth most important vital sign. It was defined by the International Association for the Study of Pain (IASP) in 2020 as an unpleasant sensory and emotional experience associated or similarly associated with tissue damage or potential tissue damage (Raja SN and Cohen, 2020). With the increase in patients’ expectations for better quality of life, the requirements for pain control are also increasing. One of the reasons patients with acute pain visit the emergency department is to resolve the pain problem quickly (Cordell et al., 2002). The principle of selecting analgesic formulations for nontraumatic soft tissue pain is to start with topical use, followed by oral route and injection (Zhang P and Guo, 2022).

The compound methyl salicylate liniment (Ammeltz) has been developed by Japan Sendai Kobayashi Pharmaceutical Co. and has been listed in Japan for many years. It is composed of various components, such as methyl salicylate, menthol, camphor, chlorpheniramine maleate, and thymol. Methyl salicylate is a common ingredient in liniments used for the relief of musculoskeletal aches and pains (Ohta et al., 2009). A patch containing methyl salicylate and menthol has been reported to provide significant relief of pain associated with mild to moderate muscle strain (Higashi et al., 2010). Camphor and menthol are naturally occurring compounds with a terpene skeleton, and are often used in topical formulations for their analgesic properties (Xu et al., 2005; Pauwels et al., 2012; Pergolizzi et al., 2018). Chlorpheniramine maleate is a classic antihistamine agent often used to relieve allergic symptoms caused by histamine (Choi et al., 2019). Thymol is a naturally occurring phenol monoterpene derivative of cymene and isomer of carvacrol, and it has antibacterial, anti-inflammatory, and antioxidant activities (Salehi et al., 2018). The indications for the use of liniment are shoulder and neck pain, low-back pain, muscle pain, or pain caused by sprains, strains, contusions, muscle swelling, and arthritis. The packaging design of Ammeltz is easy to carry and use, and the drug applied to the skin is relatively safer compared with oral drugs.

In 2009, the liniment obtained the approval from the National Medical Products Administration (Grant number: 2009L00057) for a clinical trial in China. Then, a randomized, multicenter clinical trial in China (Diclofenac sodium as positive control, six hospitals, *n* = 216) was conducted. The trial showed that Ammeltz effectively alleviated pain symptoms in patients with acute and chronic soft tissue injury, and the effective rate of pain relief was 82.24%. There were no significant changes in blood routine parameters, parameters of liver and kidney function, and urine routine parameters after the treatment. No clinically significant changes or abnormalities were found by electrocardiography. The results of the clinical trial indicated a good safety of Ammeltz, so Ammeltz was approved for listing in China in 2011.

According to the drug instructions, adverse effects of this liniment are all topical reactions, such as rash, redness, swelling, itching, pain, ulcers, and paresthesia. This multicenter, phase Ⅳ, prospective monitoring study of the drug was conducted to further observe the safety of Ammeltz in a wide range of people. And this study is the first to report the adverse drug reaction monitoring data of Ammeltz in China.

## Methods

The trial was registered in the Clinical Trial Registry (NCT05489939) and approved by the Ethics Committee of Xiangya Hospital, Central South University (Approval number: 201406063). The investigation conformed to the principles outlined in the Declaration of Helsinki, and a written informed consent was obtained from every patient involved in the clinical trial.

### Trial design and objectives

**FIGURE 1:**
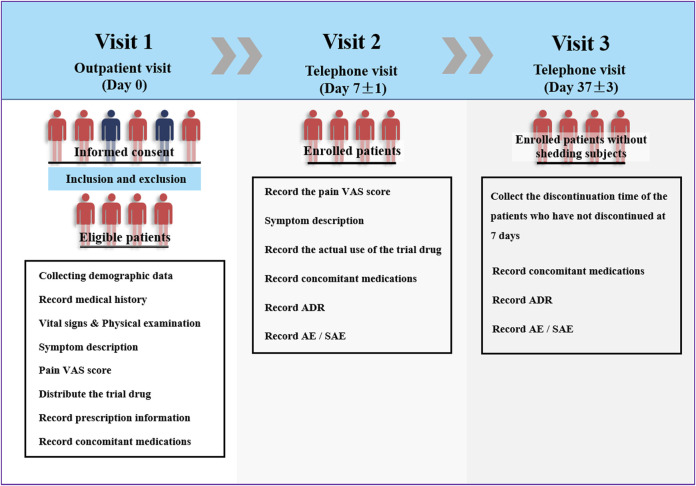
Trial design diagram.

This multicenter clinical trial was conducted at 22 hospitals/centers in China between May 2014 and March 2015. The investigators collected demographic data, vital signs, current medical history (type of soft tissue damage), and allergy history of the eligible patients on the day of Ammeltz prescription. At the same time, a doctor conducted the physical examination and evaluated the visual analog scale (VAS) score of each patient. On the seventh day of the drug administration (one course of the treatment lasts 7 days), the investigator asked each patient by telephone about the actual use of the study drug, whether adverse events occurred, and about the self-assessment pain VAS score. On the 37th day of the treatment, the patients were interviewed again by telephone to inquire about the occurrence of adverse events (Figure 1).

The primary aim of the trial was to evaluate the safety of Ammeltz in a real-life environment. The secondary monitoring objective was to evaluate the efficacy of Ammeltz in the treatment of soft tissue injury pain in a real-life environment.

### Sample size and power calculation

In principle, according to the guide for drug monitoring in production enterprises in China, the number of patients included in the statistical analysis of key drug monitoring should not be less than 3000 in order to provide ample information about the trial drug (China Food and Drug Administration: Guidelines for Drug Monitoring of Manufacturers, [2013] No.12). Therefore, the sample size of this study was chosen to be 3600 cases, considering the dropout rate of the subjects. Power analysis and the sample size were used to calculate the power of the study using PASS software (version 11, NCSS, LLC. Kaysville, Utah, United States). With a known background incidence rate of 0.0728 of a particular adverse reaction, a sample of 3,600 patients achieves 91.38% power to detect an additional incidence rate of 0.0141 when the significance level is 0.05.

### Patients selection

The inclusion criteria for the patients were as follows: 1) age 3–75 years, male or female sex; 2) soft tissue pain caused by shoulder and neck pain, back pain, muscle pain, sprain, strain, contusion, muscle swelling and pain, and arthritis; local symptoms included pain, swelling, bruising, or tenderness; 3) signed informed consent and willing to participate in the study. Patients were excluded if they met the following criteria: 1) allergy to any of the ingredients in this drug; 2) pregnant women, lactating women, and infants aged 0–3 years; 3) the affected area in the eye or mucous membranes, eczema, macules, wounds, and damaged skin; 4) patients without indications for the use of this drug.

### Obtaining informed consent

Three kinds of informed consent forms (ICF) were applied depending on the age of a subject: for those aged 3–11 years, 12–17 years, and over 18 years. For subjects under 18 years, in the company of their parents or guardians, the study doctor fully communicated with the subjects and clarified the purpose of the trial, the trial process, the possible benefits and risks, and other treatment options available, so that the subject and their parents or guardians could reach full understanding. When the subjects and their parents or guardians decided to participate in the trial, they both signed the ICF, and then the study doctor signed the ICF.

### Procedures

The procedure of the study mainly included three stages of visits (visit 1, visit 2, and visit 3). On the first visit (outpatient visit, day 0), the investigators completed the following evaluations and procedures after the patients signed the informed consent form: 1) recording the demographic data such as name, age, gender, weight, and ethnicity; 2) recording the medical history, including current medical history, diagnosis, previous medical history, and history of allergies; 3) recording vital signs; 4) performing physical examination; 5) describing symptoms; 6) training and assisting patients to determine pain VAS score; 7) distributing the trial drug and recording prescription information; 8) recording concomitant medications related to the disease.

Visit 2 (Day 7 ± 1, telephone visit) included the following: 1) recording the pain VAS score on the seventh day of medication; 2) symptom description; 3) recording the actual use of the trial drug; 4) recording concomitant medications related to the disease; 5) recording adverse drug reactions, adverse events, and serious adverse events.

Visit 3 (Day 37 ± 3, telephone visit) included the following: 1) collection of the discontinuation time of the patients who have not discontinued at 7 days; 2) recording concomitant medications related to the disease; 3) recording adverse drug reactions, adverse events, and serious adverse events.

The method of drug administration included application of an appropriate amount of the drug evenly to the affected area depending on the size of the pain area, two to four times per day.

### Safety and efficacy assessments

Adverse drug reactions, adverse events, and serious adverse events were recorded to evaluate the safety of the trial drug.

New adverse drug reactions referred to adverse reactions not previously specified in the drug label. Severe adverse drug reactions were reactions that caused one of the following outcomes: 1) death; 2) life-threatening condition; 3) cancer, deformity, and birth defects; 4) significant or permanent physical disability or impairment of organ function; 5) hospitalization or prolonged hospitalization; 6) other important medical events (the conditions listed above may occur without treatment) (International Council for Harmonisation (ICH), 2016). All of the participants were trained to report adverse reactions not only at the beginning of the clinical trial, when informed consent was obtained, but also at every visit. Data on adverse reactions were collected from self-reported symptoms and physical examination findings. The Common Terminology Criteria for Adverse Events (CTCAE, In: vol. 4.03) were used to grade adverse event severity (CTEP, 2010).

The differences in pain VAS scores before and after a course of treatment (7 days) were used to evaluate the efficacy of the trial drug. For participants under 18 years, the investigators explained to the participants and their parents or guardians how to use the VAS ruler that the study provided. Under the surveillance or with the help of their parents or guardians, the investigator and participant determined the final VAS score. The participants were given the same VAS ruler that they used at Visit 1 to use at home for Visit 2 and Visit 3. Those patients who did not complete the full 7-day treatment because of the improvement in their condition were evaluated based on the VAS score on the last treatment day. The formula for calculating the pain relief rate was as follows: Pain relief rate = (Baseline pain VAS score–The 7th-day pain VAS score)/Baseline pain VAS score × 100%. The evaluation criteria were as follows: 1) significant effect: pain relief rate ≥75%; 2) improvement: pain relief rate ≥30% and <75%; 3) inefficacy: pain relief rate <30%. Significant effect and improvement were considered effective. The baseline pain VAS score was the pain VAS score of Day 0.

### Data set

Full analysis set (FAS) was a data set of subjects who have received at least one medication and have had a baseline assessment and at least one efficacy evaluation after administration according to the basic principles of Intention-to-treat (ITT). FAS was the main population for the efficacy analysis in this study. Per-protocol set (PPS) was a data set of subjects who did not violate the protocol and who had good compliance in FAS; PPS was used as the secondary population in the efficacy analysis in this study. Safety set (SS) included all of the subjects who have received at least one study medication and have underwent at least one safety evaluation. SS was the main population in the safety analysis in this study.

Of the 3,600 subjects enrolled in this study, 27 (1.61%) did not use the study drug, and 31 (0.86%) had no medication history (lost follow-up) and did not enter the SS. A total of 3,542 patients (98.39%) entered the SS. Five patients (0.14%) who were repeatedly enrolled did not enter the FAS, and a total of 3,537 subjects (98.25%) entered the FAS. A total of 3,503 subjects (97.31%) entered PPS, whereas 15 subjects with visit time exceeding window, six subjects who used prohibited drugs (other topical medicines on the same affected area during the study period), and 13 subjects who violated the inclusion criteria one did not enter the PPS.

### Statistical analysis

Descriptive statistics (SAS 9.1.3) was used to present all data, including demographic data, efficacy evaluation indicators, and all safety data. For the measurement data, we reported the number of effective cases (missing cases), mean, standard deviation, median, lower quartile, upper quartile, minimum and maximum, and 95% confidence interval. Intragroup comparisons of the measurement data were performed using paired-samples *t*-test or paired Wilcoxon rank-sum test depending on whether the data complied with the normal distribution. For the count data, we reported their frequency and relative number (ratio, percentage, rate), and calculated 95% confidence interval of the rate. Unless otherwise specified, the hypothesis tests used in the study were two-sided tests and *p* ≤ 0.05 was considered statistically significant.

## Results

### Distribution of the subjects

A total of 3,600 subjects were enrolled in this study at 22 centers in China (Supplementary Table 1). Among them, 3,515 (97.64%) subjects completed the study, and 85 (2.36%) terminated the study early. The reasons for early termination of the study were loss to follow-up (49 cases), violation of the protocol (29 cases), other reasons (four cases did not use the study drug), voluntary termination of the study (two cases), and death (one case). The data regarding the subjects completing/terminating the study are shown in Table 1.

**TABLE 1:** Data of the subjects completing/terminating the study.

Index	Enrollment	FAS	PPS	SS
Did the subject complete the study according to the study protocol?				
Total (missing)	3600 (0)	3537 (0)	3503 (0)	3542 (0)
Yes (%)	3515 (97.64)	3515 (99.38)	3483 (99.43)	3515 (99.24)
No (%)	85 (2.36)	22 (0.62)	20 (0.57)	27 (0.76)
Loss to follow-up (%)	49 (57.65)	18 (81.82)	18 (90.00)	18 (66.67)
Protocol deviation (%)	29 (34.11)	1 (4.54)	0 (0.00)	6 (22.22)
Voluntarily terminated the study (%)	2 (2.35)	2 (9.09)	2 (10.00)	2 (7.41)
Death (%)	1 (1.18)	1 (4.55)	0 (0.00)	1 (3.70)
For safety reasons, investigators believe that discontinuation of treatment is most beneficial to the subject (%)	0 (0.00)	0 (0.00)	0 (0.00)	0 (0.00)
Pregnancy (%)	0 (0.00)	0 (0.00)	0 (0.00)	0 (0.00)
Other reasons (%)	4 (4.71)	0 (0.00)	0 (0.00)	0 (0.00)

FAS: full analysis set; PPS: Per-protocol set; SS: safety set.

### Demographic baseline characteristics

Baseline characteristics of the subjects were analyzed using the FAS (Table 2). The average age of the subjects was 45.31 ± 14.37 years (ranging from 13.82 to 89.95 years), with 1,361 male subjects (38.48%) and 2,176 female subjects (61.52%). The vast majority of the subjects (3,464 cases, 97.94%) were of Han nationality. A total of 373 (10.55%) subjects had a previous medical history, and 118 (3.35%) had a history of allergies.

**TABLE 2:** Baseline demographic characteristics of the subjects in the FAS.

Index	Value
Gender	
N (missing)	3537 (0)
Male (%)	1361 (38.48)
Female (%)	2176 (61.52)
Nationality	
N (missing)	3537 (0)
Han (%)	3464 (97.94)
Others (%)	73 (2.06)
Age (years)*	
N (missing)	3537 (0)
Mean ± SD	45.31 ± 14.36
Weight (kg)	
N (missing)	3501 (36)
Mean ± SD	62.70 ± 10.53
Past medical history	
N (missing)	3537 (0)
No (%)	3164 (89.45)
Yes (%)	373 (10.55)
History of allergies	
N (missing)	3521 (16)
No (%)	3403 (96.65)
Yes (%)	118 (3.35)
Body temperature (°C)	
N (missing)	3536 (1)
Mean ± SD	36.43 ± 0.31
Breathing (times/min)	
N (missing)	3535 (2)
Mean ± SD	18.52 ± 1.51
Heart rate (times/min)	
N (missing)	3536 (1)
Mean ± SD	74.79 ± 6.18
Systolic pressure (mm Hg)	
N (missing)	3535 (2)
Mean ± SD	120.33 ± 10.65
Diastolic pressure (mm Hg)	
N (missing)	3535 (2)
Mean ± SD	76.05 ± 7.60

FAS: full analysis set.

In the FAS, the most common diseases were muscle injury (365 cases, 10.32%), ligament sprain (365 cases, 10.32%), spinal arthropathy (353 cases, 9.98%), osteoarthritis (335 cases, 9.47%), periarthritis (332 cases, 9.39%), disc herniation (286 cases, 8.09%), arthritis (251 cases, 7.10%), fasciitis (214 cases, 6.05%), soft tissue injury (183 cases, 5.17%), and muscle strains (130 cases, 3.68%).

### Concomitant therapy

In the SS, there were 261 subjects (7.37%) who used concomitant medication (Supplementary Table 2). Of these, 219 (6.18%) subjects received a combination of medications related to the indication. The most common types of drugs were central nervous system drugs (121 subjects, 3.42%), musculoskeletal drugs (94 subjects, 2.65%), and health care products and traditional Chinese medicine (19 subjects, 0.54%). The most common drugs were diclofenac (34 subjects, 0.96%), loxoprofen (19 subjects, 0.54%), meloxicam (17 subjects, 0.48%), glucosamine (17 subjects, 0.48%), and celecoxib (14 subjects, 0.40%).

### Drug exposure

In the FAS, a total of 3,227 (91.29%) subjects completed a 7-day treatment. The subjects used 2.01 ± 0.86 administrations of drugs (Mean ± SD) on the first day, and the average of 2.24 ± 0.80 administrations on the second day, the average of 2.23 ± 0.82 administrations on the third day, the average of 2.15 ± 0.86 administrations on the fourth day, the average of 2.09 ± 0.89 administrations on the fifth day, the average of 2.00 ± 0.93 administrations on the sixth day, and the average of 1.93 ± 0.95 administrations on the seventh day. The average number of administrations of the subjects during 7 days was 2.09 ± 0.76 administrations/day (Supplementary Table 3). A total of 2,610 (74.21%) subjects continued to use the study drug after the end of one course (7 days), and 903 (34.60%) of these subjects continued using the study drug during Visit 3 (Day 37; Supplementary Table 4).

### Safety

#### Summary of adverse events

A total of 277 adverse events occurred in 258 subjects (7.28%) in the SS (Table 3). The majority of adverse events were mild, with 249 (7.03%) mild, eight (0.23%) moderate, and one (0.03%) severe. Of these, 51 cases (1.44%) were judged by the investigator to be related to the study drug (Table 3). The most common adverse events included upper respiratory infections (130 cases, 3.67%), local pruritus (17 cases, 0.48%), and diarrhea (12 cases, 0.34%). In this study, two subjects experienced serious adverse events, and in these two cases the adverse events were not related to the study drug as judged by the investigator.

**TABLE 3:** Summary of adverse events in the SS.

Adverse events		Number of cases	Incidence (%)
Total		258	7.28
Severity degree			
	Mild	249	7.03
	Moderate	8	0.23
	severe	1	0.03
Measures			
	Continued medication	145	4.09
	Dose reduction	2	0.06
	Resuming after a pause	3	0.08
	Discontinuation	42	1.19
	None	67	1.89
Relationship with the monitored drugs			
	Unrelated	209	5.90
	Related	51	1.44
Outcome			
	Death	1	0.03
	Symptoms persist	11	0.31
	Symptoms disappear without sequelae	246	6.95
Serious adverse events			
	No	256	7.23
	Yes	2	0.06

SS: safety set.

#### Summary of adverse drug reactions

Adverse drug reaction refers to an adverse reaction that occurs in a qualified drug under normal usage and has nothing to do with the purpose of the drug. A total of 50 (1.41%) subjects in the SS had 58 adverse drug reactions, and the details of adverse drug reactions are shown in Supplementary Table 5, 6. The majority of the adverse drug reactions were mild. There were 48 cases (1.36%) of mild adverse drug reactions and two (0.06%) cases were moderate. No serious adverse drug reactions occurred. The most common adverse drug reactions included local pruritus (17 cases, 0.48%), a burning sensation at the application site (10 cases, 0.28%), and irritation at the application site (local) (seven cases, 0.2%). The outcome of all adverse drug reactions was the disappearance of symptoms without sequelae, and no serious adverse drug reactions occurred (Table 4).

**TABLE 4:** Summary of adverse drug reactions in the SS.

Adverse drug reactions		Number of cases	Incidence (%)
Total		50	1.41
Severity degree			
	Mild	48	1.36
	Moderate	2	0.06
Measures		50	1.41
	Continued medication	18	0.51
	Dose reduction	2	0.06
	Resuming after a pause	3	0.08
	Discontinuation	25	0.71
	None	2	0.06
Relationship with the monitored drugs			
	May be related	45	1.27
	Definitely related	5	0.14
Outcome			
	Symptoms disappear without sequelae	50	1.41
Serious adverse events			
	No	50	1.41

SS: safety set.

A new adverse drug reaction is an adverse reaction not specified in the drug label. Among the 50 adverse drug reactions that occurred in the test subjects, no adverse reaction was determined to be a new adverse drug reaction. However, the incidence of adverse reactions in subjects over 60 years of age (2.64%) was higher than that in subjects between 18 and 60 years (1.14%).

### Efficacy

The FAS was the main population for the efficacy analysis, and the PPS was the secondary population for the efficacy analysis in this study. The change in the pain VAS score from baseline to the seventh day after treatment was used as an index of efficacy evaluation. Larger VAS scores indicated greater pain relief.

In the FAS, the VAS scores (mean ± SD) at baseline (Visit 1) and Visit 2 were 5.34 ± 1.46 and 2.79 ± 1.39, respectively. The change from Visit 2 to baseline was −2.54 ± 1.45 (*p* < 0.0001). The VAS scores (mean ± SD) at baseline (Visit 1) and Visit 2 were 5.34 ± 1.46 and 2.79 ± 1.39 in PPS, respectively. The change from Visit 2 to baseline was −2.55 ± 1.45 (*p* < 0.0001). The use of Ammeltz significantly reduced the patients’ VAS scores, which was consistent in the efficacy analyses of PPS and FAS (Table 5).

**TABLE 5:** VAS scores of the subjects.

Groups	N/VAS scores	Visit 1 (baseline)	Visit 2 (telephone follow-up)	Visit 2—visit 1	Paired test (p value)*
Total Group					
FAS	N (missing)	3537 (0)	3536 (1)	3536 (1)	−2855543 (*p* < 0.0001)
	Mean ± SD	5.34 ± 1.46	2.79 ± 1.39	−2.54 ± 1.45	
PPS	N (missing)	3503 (0)	3503 (0)	3503 (0)	−2811801 (*p* < 0.0001)
	Mean ± SD	5.34 ± 1.46	2.79 ± 1.39	−2.55 ± 1.45	
Subgroup (Subjects who used analgesics and nonsteroidal anti-inflammatory drugs were excluded)					
FAS	N (missing)	3318 (0)	3317 (1)	3317 (1)	−2524712 (*p* < 0.0001)
	Mean ± SD	5.31 ± 1.46	2.77 ± 1.38	−2.54 ± 1.44	
PPS	N (missing)	3291 (0)	3291 (0)	3291 (0)	−2491475 (*p* < 0.0001)
	Mean ± SD	5.31 ± 1.46	2.76 ± 1.38	−2.55 ± 1.44	

VAS: visual analog scale; FAS: full analysis set; PPS: Per-protocol set.

*Signed-rank test.

The subject’s pain relief rate was 47.11% ± 23.13% in the FAS. The drug was ineffective in 767 cases (21.69%), effective in 2,769 cases (78.31%), significantly effective in 439 cases (12.42%), and improved (65.89%) in 2,330 cases. In the PPS, the subject’s pain relief rate was 47.24% ± 23.09%. The drug was ineffective in 753 cases (21.50%), effective in 2,750 cases (78.50%), significantly effective in 437 cases (12.47%), and improved in 2,313 cases (66.03%). The results from the FAS and the PPS were consistent, and most of the subjects experienced pain relief after using Ammeltz (Table 6).

**TABLE 6:** Pain relief in the subjects after a course of the study drug treatment.

Groups		FAS	PPS
Total group	Pain relief rate (%)		
	N (missing)	3536 (1)	3503 (0)
	Mean ± SD	47.11 ± 23.13	47.24 ± 23.09
	Pain relief		
	Total (missing)	3536 (1)	3503 (0)
	Valid (%)	2769 (78.31)	2750 (78.50)
	Significantly effective (%)	439 (12.42)	437 (12.47)
	Improved (%)	2330 (65.89)	2313 (66.03)
	Invalid (%)	767 (21.69)	753 (21.50)

Subgroup (Subjects who used analgesics, nonsteroidal anti-inflammatory drugs were excluded)	Pain relief rate (%)		
	N (missing)	3317 (1)	3291 (0)
	Mean ± SD	47.34 ± 23.00	47.45 ± 22.97
	Pain relief		
	Total (missing)	3317 (1)	3291 (0)
	Valid (%)	2612 (78.75)	2597 (78.91)
	Significantly effective (%)	409 (12.33)	408 (12.40)
	Improved (%)	2203 (66.42)	2189 (66.51)
	Invalid (%)	705 (21.25)	694 (21.09)

FAS: full analysis set; PPS: Per-protocol set.

#### Subgroup efficacy analysis

To avoid the effects of drugs such as analgesics and nonsteroidal anti-inflammatory drugs on the evaluation of efficacy, we performed a subgroup efficacy analysis that excluded subjects using these drugs. In the FAS, the VAS scores (mean ± SD) at baseline (Visit 1) and Visit 2 were 5.31 ± 1.46 and 2.77 ± 1.38, respectively. The change in VAS scores from Visit 2 to baseline was −2.54 ± 1.44 (*p* < 0.0001). In the PPS, the VAS scores (mean ± SD) at baseline (Visit 1) and Visit 2 were 5.31 ± 1.46 and 2.76 ± 1.38, respectively. The change from Visit 2 to baseline was −2.55 ± 1.44 (*p* < 0.0001). The use of Ammeltz significantly reduced the patients’ VAS scores, which was consistent in the subgroup efficacy analyses of the PPS and the FAS (Table 5).

The subject’s pain relief rate was 47.34% ± 23.00%. The drug was ineffective in 705 cases (21.25%), effective in 2,612 cases (78.75%), significantly effective (12.33%) in 409 cases, and improved in 2,203 cases (66.42%) in the subgroup analysis of the subject’s pain relief in the FAS. In the PPS, the subject’s pain relief rate was 47.45% ± 22.97%. The treatment was ineffective in 694 cases (21.09%), effective in 2,597 cases (78.91%), significantly effective in 408 cases (12.40%), and improved in 2,189 cases (66.51%). The results of the FAS and the PPS were consistent. The majority of the subjects had pain relief after using Ammeltz (Table 6).

## Discussion

A total of 3,600 patients with soft tissue injuries were included in this study. After 7 days of treatment, the effective rate of pain relief was 78.31%. After excluding the subjects who used analgesics and nonsteroidal anti-inflammatory drugs, the pain relief rate in the subgroup reached 78.75%. These data showed that Ammeltz effectively relieved pain caused by soft tissue injury in the current real-world study.

A total of 258 subjects had adverse events, accounting for 7.28% of the SS population. Among them, 50 cases were related to Ammeltz, accounting for 1.41% of the SS population. Most of them were mild local adverse reactions, and no new adverse drug reactions occurred. Hence, Ammeltz showed good safety. We found that the incidence of adverse reactions in subjects over 60 years of age (2.64%) was higher than that in individuals aged 18–60 years (1.14%), indicating that older individuals need to pay more attention to adverse reactions when using Ammeltz. Although we found no adverse reactions in the population under 18 years of age, the safety of Ammeltz in children remains to be further explored since there were only seven individuals under the age of 18 years in our study.

Salicylates have been used to treat soft tissue pain for many years. They are similar to aspirin and nonsteroidal anti-inflammatory drugs in terms of pharmacological action, but the mechanism is not exactly the same. Skin irritation symptoms may occur when using salicylates. The incidence of adverse events reported in different studies varies widely, depending on the indications targeted and the time of administration. Mason et al. systematically reviewed the efficacy and safety of topical tinctures containing salicylates in the treatment of acute and chronic pain (Mason et al., 2004). In three double-blind placebo-controlled studies, a total of 182 patients with acute pain were included. The study found that adverse events were rare and the pain was always local. In six double-blind placebo-controlled studies, a total of 429 patients with chronic pain were included, with few adverse events reported. Derry et al. analyzed seven clinical trials of salicylate-containing topical drugs for acute pain and 10 clinical trials for chronic pain, involving a total of 1,368 patients; they found that the incidence of adverse events was 15% (range, 0%–83%), and the incidence of adverse events in the placebo group was 9% (range, 0%–52%), while with the incidence of local pain was 6% (range, 0%–24%) (Derry et al., 2014). Study has shown that the incidence of adverse reactions was 83% in 58 patients with osteoarthritis after using copper salicylate gel for 4 weeks, and the placebo group had an adverse reaction rate of 52% (Shackel et al., 1997). However, in the study by Stam et al., local adverse reactions occurred in 18 of 74 patients who used salicylic acid cream for 1 week to treat acute low-back pain, and the incidence was 24% (Stam et al., 2001). Algozzine et al. found that the use of salicylic acid cream for 1 week to treat osteoarthritis was not associated with any local adverse reactions (Algozzine et al., 1982). The above studies are summarized in Table 7.

**TABLE 7:** Adverse events of salicylates reported in different studies.

Study	Drug	N	Patients	Adverse events/Adverse event rate	Reference
Placebo-controlled study	----	182	Acute pain	Few	Mason L et al. BMJ. (2004)
Placebo-controlled study	----	429	Chronic pain	Rare	Mason L et al. BMJ. (2004)
Placebo- and active-controlled study	----	1368	Acute pain and chronic pain	15% vs 9% (control group)	Derry S et al. Cochrane Database Syst Rev. (2014)
Placebo-controlled study	Copper-salicylate gel	58	Osteoarthritis	83% vs 52% (control group)	Shackel NA et al. Med J Aust. (1997)
Active-controlled study	Cremor Capsici Compositus FNA	74	Acute low-back pain	24%	Stam C et al. Br Homeopath J. (2001)
Placebo-controlled study	10% trolamine salicylate cream	25	Symptomatic osteoarthritis	No adverse reactions	Algozzine GJ et al. JAMA. (1982)

Menthol and camphor are common in salicylate products. Menthol is classified by Food and Drug Administration (FDA) as a safe and effective topical over-the-counter (OTC) product (Kamatou et al., 2013), which has a low probability of adverse reactions. Camphor is known to be toxic (Bazzano et al., 2017) and may have some adverse effects, such as convulsions, lethargy, ataxia, severe nausea, and vomiting (Manoguerra et al., 2006). However, most of these adverse reactions are caused by gastrointestinal absorption, and even when applied to the skin in large quantities, camphor rarely causes systemic poisoning resembling the effects seen with acute ingestion exposures (Manoguerra et al., 2006). Most of the adverse reactions observed in this study were skin and application site adverse reactions, and the possibility of adverse reactions caused by these two components in this study was relatively low.

This study evaluated the safety and effectiveness of Ammeltz in a large population with a wide range of indications, and proved that Ammeltz is safe and effective in real-life use environments. Although the study obtained the expected results, there are still some limitations. Since this trial was based on a post-market safety monitoring of the study drug, the study was designed as a single arm without a control group. Therefore, it only explains the pain relief through comparison of the conditions before and after the medication. However, interference factors such as disease progression cannot be ruled out in the evaluation of the efficacy.

## Conclusion

Ammeltz is safe and effective in real-life applications. It can significantly relieve soft tissue pain caused by shoulder and neck pain, back pain, muscle pain, and other causes. No new adverse drug reactions were found in our real-world study.

## Data Availability

The original contributions presented in the study are included in the article/Supplementary Material, further inquiries can be directed to the corresponding author.
