# Supplementary material for: Safety and efficacy of compound methyl salicylate liniment for topical pain: A multicenter real-world study in China
Source: Front Pharmacol. 2022 Oct 21;13:1015941. doi: 10.3389/fphar.2022.1015941 (PMC9634125; doi:10.3389/fphar.2022.1015941)
Supplement: Supplementary file 1 [file DataSheet1.PDF]

Supplementary Table 1 Cases distribution.

Centers	Enrollment	FAS	PPS	SS
Beijing Hospital	250	246	245	246
The Fourth People's Hospital of Sichuan Province	120	120	120	120
Chengdu Second People's Hospital	100	100	100	100
Affiliated Hospital of Chengdu University of Traditional Chinese Medicine	100	97	94	97
Xi 'An Second Hospital	187	187	184	187
Xi 'An Honghui Hospital	120	120	120	120
The Fourth People's Hospital of Shaanxi Province	53	53	53	53
Urumqi Hospital of Traditional Chinese Medicine	120	120	120	120
Xinjiang Production and Construction Corps Hospital	100	100	100	100
The First Affiliated Hospital of Hunan University of Traditional Chinese Medicine	150	140	137	141
Hunan Provincial People's Hospital	350	324	313	324
Xiangya Hospital Central South University	200	185	183	189
Shanghai Xuhui District Central Hospital	50	49	47	49
Shuguang Hospital Affiliated to Shanghai University of Chinese Medicine	200	198	196	198
Shanghai Hospital of Integrated Traditional Chinese and Western Medicine	150	149	145	149
Nanfang Hospital, Southern Medical University	300	300	298	300
People's Liberation Army No. 202 Hospital	200	200	200	200
The Second Affiliated Hospital of Liaoning University of Traditional Chinese Medicine	210	210	210	210
The First Affiliated Hospital of Heilongjiang University of Traditional Chinese Medicine	100	100	100	100
Qingdao Haici Medical Group	230	230	230	230
The Second Affiliated Hospital of Nanjing Medical University	150	150	150	150
Suzhou Tongji Hospital	160	159	158	159
**Total**	3600	3537	3503	3542

FAS: Full analysis set; PPS: Per-protocol set; SS: Safety set.

Supplementary Table 2 Combined medication.

Index	N (%)
Combined medication	
Total (missing)	3542 (0)
No (%)	3281 (92.63)
Yes (%)	261 (7.37)

Supplementary Table 3 Detailed use of the 7-day study drug.

	N	FAS
Whether the patient complete the 7-day treatment with the study drug?		
Total (missing)		3535 (2)
No (%)		308 (8.71)
Yes (%)		3227 (91.29)
Medication Day 1	N (missing)	3537 (0)
	Mean ± SD	2.01 ± 0.86
Medication Day 2	N (missing)	3537 (0)
	Mean ± SD	2.24 ± 0.80
Medication Day 3	N (missing)	3537 (0)
	Mean ± SD	2.23 ± 0.82
Medication Day 4	N (missing)	3537 (0)
	Mean ± SD	2.15 ± 0.86
Medication Day 5	N (missing)	3537 (0)
	Mean ± SD	2.09 ± 0.89
Medication Day 6	N (missing)	3536 (1)
	Mean ± SD	2.00 ± 0.93
Medication Day 7	N (missing)	3503 (34)
	Mean ± SD	1.93 ± 0.95
Average number of administrations	Mean ± SD	2.09 ± 0.76

Supplementary Table 4 Continuing the use of the study drug.

N	FAS
Is Visit 2 discontinued?	
Total (missing)	3517 (20)
No (%)	2610 (74.21)
Yes (%)	907 (25.79)
Is Visit 3 discontinued?	
Total (missing)	2610 (0)
No (%)	903 (34.60)
Yes (%)	1707 (65.40)

Supplementary Table 5 The details of adverse drug reactions.

Adverse drug reactions	Number of cases	Incidence (%)
**Total**	50	1.41
**Systemic disease and administration site reactions**	26	0.73
Burning sensation at the application site	10	0.28
Application site (local) irritation	7	0.2
Localized transient papules	5	0.14
Paresthesia at the application site	5	0.14
Pain at the application site	3	0.08
Desquamation of skin at the application site	2	0.06
**Skin and subcutaneous tissue diseases**	23	0.65
Local itching	17	0.48
Local skin redness	5	0.14
Dermatitis	1	0.03
**Immune system disease**	2	0.06
Local mucous membrane irritation of the eye	1	0.03
Drug allergy	1	0.03
**Various musculoskeletal and connective tissue disorders**	1	0.03
Musculoskeletal pain	1	0.03

Supplementary Table 6 Frequency analysis of adverse drug reactions by age

ADR	Number of cases	Frequency	Incidence (%)
18 ≤age ≤60 years	33	40	1.14
Local itching	11	11	0.38
Burning sensation at the application site	6	6	0.21
Local skin redness	5	5	0.17
Localized transient papules	5	5	0.17
Application site (local) irritation	4	4	0.14
Pain at the medication site	3	3	0.1
Paresthesia at the application site	2	2	0.07
Musculoskeletal pain	1	1	0.03
Dermatitis	1	1	0.03
Local mucous membrane irritation of the eye	1	1	0.03
Skin scaling at the medication site	1	1	0.03
Age >60 years	17	18	2.64
Local itching	6	6	0.93
Burning sensation at the application site	4	4	0.62
Application site (local) irritation	3	3	0.47
Paresthesia at the application site	3	3	0.47
Drug allergy	1	1	0.16
Skin scaling at the medication site	1	1	0.16
